# Genome Sequence of *Enorma* sp. Strain Marseille-P9525^T^, a Member of a Human Gut Microbiome

**DOI:** 10.1128/MRA.00785-19

**Published:** 2019-10-10

**Authors:** Nina Gouba, Jamal Saad, Michel Drancourt, Mustapha Fellag

**Affiliations:** a Université Nazi Boni, UFR-ST Département Sciences Biologiques, Bobo-Dioulasso, Burkina Faso; b IHU Méditerranée Infection, Marseille, France; c Aix-Marseille University, IRD, MEPHI, IHU Méditerranée Infection, Marseille, France; Indiana University, Bloomington

## Abstract

Strain Marseille-P9525^T^ was isolated from the gut microbiota of a 28-year-old woman and exhibits a 2.23-Mb (G+C content, 66.8%) draft genome sequence containing 1,902 protein-coding genes, 49 tRNAs, and 3 rRNAs. The 16S rRNA sequencing and *in silico* DNA-DNA hybridization indicated that strain Marseille-P9525^T^ represents a new species to be described.

## ANNOUNCEMENT

Enorma massiliensis was the first species of the genus *Enorma* recently described within the family *Coriobacteriaceae* (1). The genus *Enorma* contains three species, namely, *E. massiliensis,* Enorma timonensis, and Enorma phocaeensis, which are all Gram-positive, obligatory anaerobic, and nonmotile bacteria isolated from human fecal microbiota under an anaerobic atmosphere (1–3). A culture-based study (4) of the stools from a 28-year-old healthy woman living in Burkina Faso yielded the isolation and culture of strain Marseille-P9525^T^ (CSUR P9525). Here, we report the genome sequence of strain Marseille-P9525^T^ and describe its genomic content.

Strain Marseille-P9525^T^ was subcultured on Colombia agar incorporating 5% sheep blood (bioMérieux, Marcy l’Etoile, France) at 37°C under anaerobic conditions (AnaeroGen Compact; Oxoid, Thermo Scientific, Dardilly, France). DNA was extracted in 50 μl using the EZ1 BioRobot and EZ1 DNA tissue kit (Qiagen, Courtaboeuf, France) and was quantified using a Qubit assay with a high-sensitivity kit (Life Technologies, Carlsbad, CA, USA) to 0.2 ng/liter. DNA was then sequenced using MiSeq technology (Illumina, Inc., San Diego, CA, USA) with paired-end applications. The DNA was fragmented and amplified by limited PCR (12 cycles), introducing dual-index barcodes and sequencing adapters. After purification on AMPure XP beads (Beckman Coulter, Inc., Fullerton, CA, USA), libraries were normalized and pooled for MiSeq paired-end sequencing. An automated cluster generation of 12 runs with dual-indexed 2 × 251-bp reads was performed for 40 hours. A total of 7,810,996 paired-end reads with a 35 to 251-bp sequence length were quality checked using FastQC, trimmed using Trimmomatic version 0.36.6 (5), and assembled using SPAdes software version 3.5.0 (6). To run SPAdes, we used the pipeline option “–careful” in order to reduce the number of mismatches and short indels. Default parameters for k values, i.e., k-mer values of 127, 99, 77, 55, 33, and 21, were applied, and default parameters were used for all software, unless otherwise noted. Finally, the Marseille-P9525^T^ draft genome was assembled into 48 contigs and 48 scaffolds, with a total size of 2,234,108 bp and a G+C content of 66.8%, giving an unclosed molecule. Automatic annotation was performed with Prokka version 1.12 (7). The genome (one chromosome but no plasmid) presents one repeat region and is predicted to carry 1,951 genes, including 1,902 protein-coding genes and 49 tRNA genes. Strain Marseille-P9525^T^ exhibits 98.3% 16S rRNA gene sequence identity with *E. phocaeensis* strain Marseille-P3242 (GenBank accession number NR_147403), 96.76% identity with *E. timonensis* (NR_144707), 95.30% with *E. massiliensis* (NR_125606), 94.03% with Collinsella tanakaei (NR_112899), and 93.96% with Collinsella ihuae (LN881598).

The 2.23-Mb draft genome sequence of strain Marseille-P9525^T^ is smaller than those *of E. phocaeensis*, *E. massiliensis*, *E. timonensis*, *C. tanakaei*, and *C. ihuae* (2.38 Mb, 2.37 Mb, 2.33 Mb, 2.49 Mb, and 2.84 Mb, respectively). Based on the 16S rRNA gene sequence proximity, genomes were selected and incorporated into an *in silico* genomic comparison. Genomic similarities estimated using OrthoANI version 0.93.1 (https://www.ezbiocloud.net/tools/orthoani) and *in silico* DNA-DNA hybridization (http://ggdc.dsmz.de/ggdc.php#) estimated using the GGDC version 2.0 online tool yielded 84.20% and 28.4% sequence similarity with *E. phocaeensis*, 80.77% and 25.3% with *E. timonensis*, 78.68% and 24.9% with *E. massiliensis*, 77.35% and 23.7% with *C. ihuae*, and 74.57% and 22.2% with *C. tanakaei*, respectively.

### Data availability.

The genome sequence reported here and the raw reads have been deposited at EMBL/GenBank under the accession numbers CABFVT000000000 and ERR3427549, respectively.

## References

[1] Mishra AK, Hugon P, Lagier JC, Nguyen TT, Couderc C, Raoult D, Fournier PE 2013 Non contiguous-finished genome sequence and description of *Enorma massiliensis* gen. nov., sp. nov., a new member of the family *Coriobacteriaceae*. Stand Genomic Sci 8:290–305. doi:10.4056/sigs.3426906. 23991260 PMC3746427

[2] Ramasamy D, Dubourg G, Robert C, Caputo A, Papazian L, Raoult D, Fournier PE 2014 Non contiguous-finished genome sequence and description of *Enorma timonensis* sp. nov. Stand Genomic Sci 9:970–986. 25197477 10.4056/sigs.4878632 PMC4149031

[3] Ndongo S, Cadoret F, Dubourg G, Delerce J, Fournier PE, Raoult D, Lagier JC 2017 “*Collinsella phocaeensis*” sp. nov., “*Clostridium merdae*” sp. nov., “*Sutterella massiliensis*” sp. nov., “*Sutturella timonensis*” sp. nov., “*Enorma phocaeensis*” sp. nov., “*Mailhella massiliensis*” gen. nov., sp. nov., “*Mordavella massiliensis*” gen. nov., sp. nov. and “*Massiliprevotella massiliensis*” gen. nov., sp. nov., 9 new species isolated from fresh stool samples of healthy French patients. New Microbes New Infect 17:89–95. doi:10.1016/j.nmni.2017.02.005. 28409003 PMC5382032

[4] Lagier JC, Hugon P, Khelaifia S, Fournier PE, Scola BL, Raoult D 2015 The rebirth of culture in microbiology through the example of culturomics to study human gut microbiota. Clin Microbiol Rev 28:237–264. doi:10.1128/CMR.00014-14. 25567229 PMC4284300

[5] Bolger AM, Lohse M, Usadel B 2014 Trimmomatic: a flexible trimmer for Illumina sequence data. Bioinformatics 30:2114–2120. doi:10.1093/bioinformatics/btu170. 24695404 PMC4103590

[6] Bankevich A, Nurk S, Antipov D, Gurevich AA, Dvorkin M, Kulikov AS, Lesin VM, Nikolenko SI, Pham S, Prjibelski AD, Pyshkin AV, Sirotkin AV, Vyahhi N, Tesler G, Alekseyev MA, Pevzner PA 2012 SPAdes: a new genome assembly algorithm and its applications to single-cell sequencing. J Comput Biol 19:455–477. doi:10.1089/cmb.2012.0021. 22506599 PMC3342519

[7] Seemann T 2014 Prokka: rapid prokaryotic genome annotation. Bioinformatics 30:2068–2069. doi:10.1093/bioinformatics/btu153. 24642063

